# An Editing-Site-Specific PCR Method for Detection and Quantification of *CAO1*-Edited Rice

**DOI:** 10.3390/foods10061209

**Published:** 2021-05-27

**Authors:** Hongwen Zhang, Jun Li, Shengbo Zhao, Xiaohong Yan, Nengwu Si, Hongfei Gao, Yunjing Li, Shanshan Zhai, Fang Xiao, Gang Wu, Yuhua Wu

**Affiliations:** Key Laboratory of Biology and Genetic Improvement of Oil Crops of the Ministry of Agriculture and Rural Affairs, Oil Crops Research Institute, Chinese Academy of Agricultural Sciences, Wuhan 430062, China; zhanghongwen_z@163.com (H.Z.); lijuner@caas.cn (J.L.); shengbo_ab@163.com (S.Z.); yanxiaohong@caas.cn (X.Y.); snw_xssnnn@163.com (N.S.); gaohongfei@caas.cn (H.G.); liyunjing@caas.cn (Y.L.); zhaishanshan@caas.cn (S.Z.); xf_hzau@163.com (F.X.); wugang@caas.cn (G.W.)

**Keywords:** genome-edited plants, *CAO1*-edited rice, editing-site-specific PCR, identification, quantification

## Abstract

Genome-edited plants created by genome editing technology have been approved for commercialization. Due to molecular characteristics that differ from classic genetically modified organisms (GMOs), establishing regulation-compliant analytical methods for identification and quantification of genome-edited plants has always been regarded as a challenging task. An editing-site-specific PCR method was developed based on the unique edited sequence in *CAO1*-edited rice plants. Test results of seven primer/probe sets indicated that this method can identify specific *CAO1*-edited rice from other *CAO1*-edited rice and wild types of rice with high specificity and sensitivity. The use of LNA (locked nucleic acid) in a probe can efficiently increase the specificity of the editing-site-specific PCR method at increased annealing temperature which can eliminate non-specific amplification of the non-target. The genome-edited ingredient content in blinded samples at the level of 0.1% to 5.0% was accurately quantified by this method on the ddPCR platform with RSD of <15% and bias in the range of ±17%, meeting the performance requirements for GMO detection method. The developed editing-site-specific PCR method presents a promising detection and quantification technique for genome-edited plants with known edited sequence.

## 1. Introduction

Genome editing technology is commencing a new crop improvement era through fast and accurate editing of target genes. Genome editing technology applies the sequence-specific nucleases (SSNs), such as zinc-finger nucleases (ZFNs), transcriptional activator-effector nucleases (TALENs) and CRISPR-related endonuclease Cas9 (CRISP/Cas9), to create double-stranded breaks (DSBs) in target genes, which are repaired through the error-prone non-homologous end-joining (NHEJ) pathway or homology-directed repair (HDR) pathway [1,2,3]. Repairing DSBs randomly introduces base insertions, deletions, or substitutions which usually result in mutations of gene knockout, gene knock-in, or gene replacement at a specific site of the genome [4,5]. Genome editing techniques have been successfully implemented to modify a specific gene/locus in many cereal crops of importance such as powdery mildew-resistant wheat and bacterial blight-resistant rice as well as in other plants such as herbicide-resistant tobacco and extended shelf-life tomato [6,7,8,9,10]. Several genome-edited plants have been approved for commercialization and consumption, for instance an herbicide-tolerant canola variety and a soybean variety with modified oil composition have been commercialized in North America [11], while a high GABA tomato variety developed using CRISPR/Cas9 has been approved for cultivation and consumption in Japan [12].

Genome editing techniques mostly introduce a knockout mutation in the endogenous target gene in the form of a short deletion or insertion, even a single-nucleotide deletion or insertion, which makes the genome-edited plants indistinguishable in molecular characteristics from those developed through mutagenesis techniques and natural mutation [13,14,15]. Since the genome-edited plants are different from classic genetically modified organisms (GMOs) harboring exogenous regulatory elements and functional genes, the discussion on how to regulate genome-edited plants is ongoing, and the regulation regarding genome-edited plants is currently not well-defined globally. The European Union (EU) Court of Justice (ECJ) declared that genome-edited crops should be as strictly regulated as GMOs in 2018 [16] and New Zealand has taken the same stance as the EU [17]. However, in America and Japan, genome-edited crops are not considered to be GMOs if the crops do not contain foreign genes [15,18]. In China, plants obtained by genome editing techniques fall within the scope of GMOs since their genetic material has been altered using gene engineering technology, meaning that genome-edited plants are regulated according to the existing GMO regulations. The surveillance of genome-edited plants requires the development of regulation-compliant identification and quantification methods for genome-edited plants.

Compared to the molecular characteristics of classic GMOs, the genome-edited crops lack foreign regulatory elements and foreign genes. The detection methods for classic GMOs that are based on the amplification of foreign DNA, involving element-specific, construct-specific, or event-specific methods, are not suitable for detecting genome-edited crops. Major progress has been made in developing analytical methods to detect genome-edited plants, such as T7E1 [19], genome editing test PCR [20], annealing at critical temperature PCR [21], oligoribonucleotide interference-PCR [22], quantitative real-time PCR coupled with high resolution melting analysis [23], gene-editing frequency digital PCR [24], quantitative real-time (qPCR)-based method [25], and single-strand conformational polymorphism [26]. The above methods only apply for the detection of a known target gene, and most of the developed methods could determine whether the target gene has been edited in a genome or evaluate genome editing efficiency, but they fail to identify and quantify the specific edited sequence contained in the genome-edited plants. Identification of classic GMOs is achieved by an event-specific detection method that is developed based on the unique junction sequence between insert DNA and flanking genomic DNA. Similar to the detection of classic GMOs, a method for the detection of a genome-edited plant, should also allow its required identification [27], so the detection results can unambiguously differentiate a specific genome-edited product from others. An herbicide-tolerant canola harboring a single-nucleotide genome edit (SU Canola, Cibus US LLC/FalcoBrand, San Diego, CA, USA) is the first commercialized genome-edited crop, and a qPCR method, using primers that carry locked nucleic acids (LNAs), was successfully developed to detect and quantify the single base pair edit canola [11].

According to the definition of GMOs, genome-edited plants are considered to be GMOs and the existing regulatory frameworks for GMOs may apply to the traceability, labeling, and monitoring of genome-edited plants [27,28]. The enforcement of GMO regulation frameworks is based on detection. Similar to the classic GM plants, methods compliant with regulations must be developed to be able to accurately identify and quantify the specific genome-edited product. Identification and quantification of genome-edited plants is regarded as difficult work, and single-nucleotide mutations are the most challenging type of genome edit [27]. The *chlorophyllide a oxygenase 1* (*CAO1*) gene regulating the production of chlorophyll in rice, was edited by using gene editing technology in our laboratory, and seven *CAO1*-edited homologous plants were identified to contain deletions or insertions of 1–18 base pairs (bps). To address this challenge, *CAO1*-edited rice plants available to us were used as materials to develop an editing-site-specific PCR method on the basis of the uniqueness of the edited sequence. We hope that this research will provide a reference for developing a GMO regulation-compliant method for the surveillance of genome-edited plants.

## 2. Materials and Methods

### 2.1. Materials

**Figure 1:**
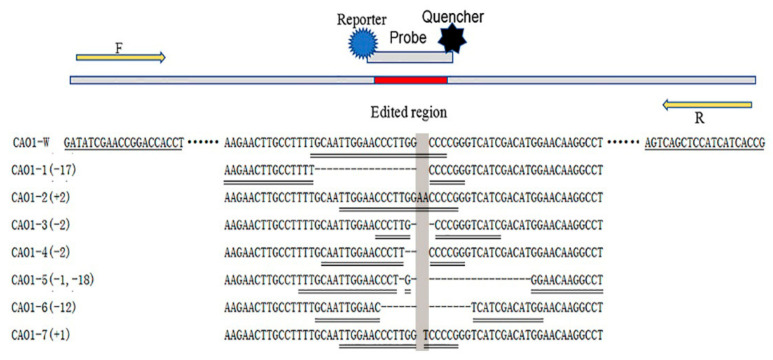
Schematic diagram of primer/probe design. Either deletions or insertions happened to the *CAO1*-edited rice (CAO1-1 ~ CAO1-7), the symbol “-” shows deleted bases and the shadow shows inserted bases. A universal primer pair (CAO1-F/R) is underlined by single line, the universal primers located upstream and downstream of edited region of *CAO1* gene. The TaqMan probes are underlined by double line, eight probes are designed to target at the edited region of wild type DNA and edited type DNA, respectively.

The genome-edited rice was obtained by using a CRISPR/Cas9 system to edit the *CAO1* gene in Nipponbare (*Oryza sativa* L. *japonica*). Seven *CAO1*-edited homozygous plants, named CAO1-1 ~ CAO1-7, were identified by Sanger sequencing and used as materials for developing analytical methods of genome-edited plants. The molecular characteristics of the edited-type and wild-type rice are shown in Figure 1: CAO1-1 rice harbors a deletion of 17 bps, the CAO1-2 rice harbors a 2 bp insertion, CAO1-3 and CAO1-4 rice both carry a 2 bp deletion, CAO1-5 rice carries a single-nucleotide deletion and a 18 bp deletion, CAO1-6 rice carries a 12 bp deletion, CAO1-7 rice only contain a single-nucleotide insertion. Nipponbare is used as a wild type control.

### 2.2. Primers and TaqMan Probes

Primer 5.0 software was used to design forward primers and reverse primers that were located upstream and downstream of the edited region of *CAO1* gene, respectively. Beacon Designer 8.0 software was used to design editing-site-specific TaqMan probes that matched with the edited sequences of the edited type rice and the wild type rice. The *PLD* (*Phospholipase D*) gene was selected as the rice reference gene in this study [29]. The primers and probes were synthesized by Sangon Biotech (Sangon, Shanghai, China). The edited type probes (CAO1-P_1_ ~ CAO1-P_7_) were labeled with 6-carbocyfluorescein (FAM) at the 5′ end and BHQ-1 at the 3′end, and the probe CAO1-P_7_ of CAO1-7 rice carried a single—Thymine (T) insertion which was labeled as locked nucleic acid (LNA). The probes of the wild type rice (CAO1-P_w_) were labeled with two different fluorophores, FAM and hexachlorofluorescein (HEX), at the 5′ end and BHQ-1 at the 3′end. The sequences of primers and probes together with other associated information are listed in Table 1.

### 2.3. DNA Extraction

Wild type and CAO1-edited rice were grown in a greenhouse that simulates rice growing conditions. Fresh leaves of wild type and *CAO1*-edited rice were collected for DNA extraction. The NuClean Plant Genomic DNA kit (CWBIO, Beijing, China) was used to extract genomic DNA from leaf powder following the kit instructions. The extracted DNA was diluted in a 0.1 × TE buffer, then the OD (optical density) values at 230 nm, 260 nm and 280 nm of DNA solution were measured using NanoDrop 2000 (Thermo Scientific, MA, USA) to evaluate the quantity and quality of genomic DNA. All extracted DNA was adjusted to a concentration of about 100 ng/μL and stored at −20 °C for subsequent analysis.

### 2.4. Preparation of Blinded Samples

The copy number concentration of genomic DNA was measured using the *PLD* droplet digital PCR (ddPCR) method [30,31]. The blinded samples were prepared by mixing edited type DNA and wild type DNA using a gravimetric method. A calibrated balance was used to weigh edited type DNA and wild type DNA, which were mixed together to prepare a copy number ratio of 5% DNA solution, then the 5% DNA solution was further diluted by adding wild type DNA stock solution to prepare a 2% DNA solution. Using the same strategy, the 2% DNA solution was further diluted by adding wild type DNA stock solution to prepare 1% DNA solution, and 1% DNA solution was used to prepare 0.1% DNA solution. A total of five sets of blinded samples were prepared by mixing the wild type DNA and the five edited types of DNA for CAO1-2, CAO1-3, CAO1-4, CAO1-5, and CAO1-7, respectively. Each set of blinded samples included four levels of 5%, 2%, 1%, and 0.1%.

### 2.5. Real-Time PCR

The real-time PCR assays were all performed on the CFX96 Touch Deep Well Real-Time PCR System (Bio-Rad, Hercules, CA, USA) in a final volume of 20 μL. The reaction mixture of SYBR Green real-time PCR contained 10 μL of 2× iTaqTM Universal SYBR Green Supermix (Bio-rad), 0.8 μL of each primer (10 μM), 1 μL of template DNA, and a final addition of ddH_2_O to 20 μL. The PCR program followed these steps: 95 °C for 15 min denaturation; 50 cycles of 95 °C for 10 s, 60 °C for 30 s, 72 °C for 30 s; then a melting curve ranging from 65 °C to 95 °C at 0.5 °C/s. The reaction mixture of TaqMan real-time PCR included 10 μL of 2× Heiff UNICON^®^ qPCR TaqMan Probe Master Mix (Yeason, Shanghai, China), 0.8 μL of each primer (10 μM), 0.4 μL of TaqMan Probe (10 μM), 1 μL template DNA, and a final addition of ddH_2_O to 20 μL. The TaqMan real-time PCR was performed following this program: 95 °C for 1 min, 45 cycles of 95 °C for 10 s, 60 °C for 60 s. The temperature of annealing and extension was increased to 66.8 °C when the LNA-containing probe was used for real-time PCR. The final data were collected using Bio-Rad CFX Manager software (v3.1.1517.0823).

### 2.6. Droplet Digital PCR

Droplet digital PCR (ddPCR) assays were performed using the QX200 system (Bio-Rad, Hercules, CA, USA) in a 20 μL reaction mixture that included 10 μL of 2× ddPCR Supermix (Bio-Rad), 0.8 μL of each primer (10 μM), 0.4 μL of probe (10 μM), 1 μL of template DNA, and a final addition of ddH_2_O to 20 μL. A total of 20 μL of mixture together with 70 μL of DG oil was added into a Bio-rad DG8^TM^ cartridge, which was placed into a QX200 Droplet Generator to generate water-in-oil droplets. 40 μL of droplets was transferred into the 96-well plate to perform PCR amplification on a C1000 Touch ^TM^ thermal cycler (Bio-Rad, Hercules, CA, USA) following this program: 95 °C for 10 min; 45 cycles of 94 °C for 30 s, (55–62 °C) for 1 min; 98 °C for 10 min; final cooling to 4 °C. After amplification, the 96-well plate was loaded onto a QX200 droplet reader (Bio-rad, Hercules, CA, USA) to read droplets using Bio-rad QuantaSoft^TM^ software (v1.7.4.0917). The default setting for thresholds was initially adopted to allow the distinction between positive droplets and negative droplets. If the default setting failed to work, the threshold was manually set up to analyze droplets.

### 2.7. Estimation of Genome-Edited Ingredient Content

Both real-time quantitative PCR (qPCR) and ddPCR can be used to estimate the content of a genome-edited ingredient; the rice reference gene *PLD*, a single-copy gene in the rice haploid, was used to measure the copy number of total genomic rice DNA [29] (Li et al., 2015). For qPCR, the serially diluted genomic DNA with known copy number concentrations from homozygous genome-edited plants was used to construct standard curves of genome-edited DNA and the *PLD* gene before the copy numbers of genome-edited DNA and the *PLD* gene in testing samples were calculated on the basis of the constructed standard curves and measured Ct values, respectively. For ddPCR, the copy number of genome-edited DNA and the *PLD* gene in testing samples can be directly obtained without a need for reference materials. In the case of using the *PLD* gene to quantify the total rice DNA, the content of genome-edited ingredient (C_E_) can be calculated to be: C_E_ (%) = copy number of edited type DNA/copy number of *PLD* gene × 100. This method of calculation is commonly adopted to estimate GMO content.

In this study, the methods quantifying both edited type DNA and corresponding wild type DNA were developed, the sum of the copy number of genome-edited DNA and wild type DNA is theoretically equivalent to the copy number of total DNA measured by the *PLD* gene method. Therefore, we can achieve measurement of the copy number of total rice DNA by quantifying the copy number of edited type DNA and wild type DNA by ddPCR; in this case, the content of genome-edited ingredient (C_E_) can be calculated to be: C_E_ (%) = copy number of edited type DNA/(copy number of edited type DNA + copy number of wild type DNA) × 100. The feasibility of this calculation method was tested by comparing it to the previously mentioned method.

## 3. Results and Discussion

### 3.1. Design of an Editing-Site-Specific PCR Method

The *CAO1*-edited rice plants carry deletions or insertions of 1-18 bps in their genome (Figure 1). To uniquely distinguish each *CAO1*-edited plant from other *CAO1*-edited rice and wild type rice, we proposed an editing-site-specific PCR method, in which a pair of universal primers was designed to amplify an amplicon spanning the edited region of *CAO1* gene and a set of TaqMan probes annealing to the edited sequences was designed to specifically identify each CAO1-edited plant (Figure 1). Similar to the event-specific methods of classic GMOs, the editing-site-specific PCR method applied a primer pair as well as an editing-site-specific TaqMan probe to achieve specific detection of genome-edited plants. It was expected that this method could identify and quantify the corresponding *CAO1*-edited rice with enough specificity and accuracy.

### 3.2. Specificity of the Editing-Site-Specific PCR Method

The selection of a universal primer pair is critical for the sensitivity of the PCR method. Three primer pairs were designed and submitted to screening of the optimal primer pair by the SYBR Green real-time PCR (Supplementary Table S1). According to the fluorescent signal intensity of amplification plots and the uniqueness of melting curve peaks, the primer pair CAO1-F1/R1, displaying the lowest Ct value and a unique melting curve peak, was selected as the primary candidate primer pair (Supplementary Figure S1). Then, using the serially diluted DNA from Nipponbare (1 copy—10^5^ copies) as templates, the SYBR Green real-time PCR was further performed to evaluate the amplification efficiency and sensitivity of the candidate primer pair. The amplification efficiency was estimated to be 104% (Supplementary Figure S1) and the lowest DNA amount that this primer pair could detect was less than 10 copies, meeting the acceptance criteria of PCR method. The primer pair CAO1-F1/R1 (renamed CAO1-F/R) was fit for subsequent analysis by combining with each edited type probe and wild type probe.

**Figure 2:**
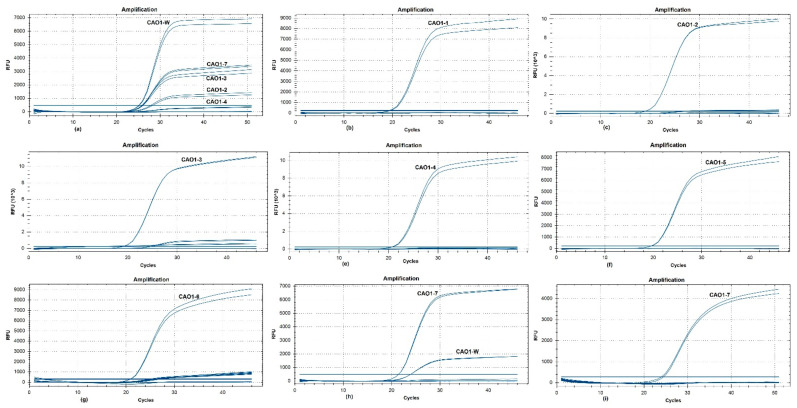
Specificity of eight primer/probe sets with wild type DNA and edited type DNA as templates. (**a**) the amplification curves of primer/probe set of CAO1-F/R/P_W_; (**b**) the amplification curves of CAO1-F/R/P_1_; (**c**) the amplification curves of CAO1-F/R/P_2_; (**d**) the amplification curves of CAO1-F/R/P_3_; (**e**) the amplification curves of CAO1-F/R/P_4_; (**f**) the amplification curves of CAO1-F/R/P_5_; (**g**) the amplification curves of CAO1-F/R/P_6_; (**h**) the amplification curves of CAO1-F/R/P_7_; (**i**) the amplification curves of CAO1-F/R/LNA-containing P_7_.

The primer set CAO1-F/R was sequentially combined with the wild type probe (CAO1-P_w_) and seven edited type probes (CAO1-P_1_ ~ CAO1-P_7_) to perform real-time PCR for evaluating the specificity of each primer/probe combination, using the genomic DNA from wild type rice and *CAO1*-edited rice as templates. The amplification plots are shown in Figure 2. The primer/probe set of CAO1-F/R/P_w_ generated typical amplification curves from wild type DNA, and amplification curves with relatively weak signal were also observed from the edited type DNA of CAO1-2, CAO1-3, CAO1-4, and CAO1-7 (Figure 2a). Sequence alignment revealed only one or two base differences at the nucleotide level between wild type rice and the four edited rice plants (CAO1-2, CAO1-3, CAO1-4, and CAO1-7) (Figure 1), the minor differences in sequences resulted in unspecific amplification signal of the CAO1-F/R/P_w_. The edited type sets of CAO1-F/R/P_1_, CAO1-F/R/P_2_, CAO1-F/R/P_3,_ CAO1-F/R/P_4_, CAO1-F/R/P_5_, and CAO1-F/R/P_6_, all yielded expected amplification curves from corresponding edited type DNA (Figure 2b–g). Although the two sets of CAO1-F/R/P_3_ and CAO1-F/R/P_6_ produced weak and unspecific amplification signals, this would not affect the judgement of testing results because of the significant difference in signal intensity between specific and unspecific amplification (Figure 2d,g).

The CAO1-7 rice is a single-nucleotide variant plant compared to the wild type rice Nipponbare. The ordinary probe of CAO1-7 rice (CAO1-P_7_) containing a single-nucleotide variation generated amplification curves from both CAO1-7 and wild type rice (Figure 2h). Chhalliyil et al. developed a PCR method specific to the commercial single-nucleotide editing of SU canola by the use of LNAs in primer design; the use of LNAs was demonstrated to be able to efficiently increase the specificity of the primer/probe system [11]. The strategy of LNAs was adopted in CAO1-P_7_ probe design, and the inserted Thymine (T) in CAO1-P_7_ probe was labeled as LNA (Table 1). The LNA-containing probe CAO1-P_7_ was combined with the primer pair CAO1-F/R to perform real-time PCR at an increased annealing temperature of 66.8 °C. This primer/probe set only generated expected signal curves from CAO1-7 DNA (Figure 2i). The application of LNA in probe and increased annealing temperature eliminated non-specific amplification of wild type DNA. Although the probe of wild type rice produced unspecific amplification from the four edited type DNA strands, the seven probes, designed to target edited sequences, can differentiate the specific edited type DNA from other edited type DNA and wild type DNA, thereby exhibiting high specificity.

### 3.3. Sensitivity of the Editing-Site-Specific PCR Method

The wild type DNA and edited type DNA were serially diluted to 80, 40, 20, 10, 5, and 1 copies/μL, respectively. The serial dilutions were used as templates to evaluate the detection sensitivity of eight primer/probe sets. The templates of ≥10 copies were set four parallels, and the templates of ≤5 copies were set ten parallels. The sensitivity test results are summarized in Supplementary Table S2. For all primer/probe sets only one or two reactions generated positive signals when the template amount decreased to one copy. Ten reactions of five primer/probe sets all generated positive signals when the template amount decreased to five copies, and the detection sensitivity of five sets of CAO1-F/R/P_w_, CAO1-F/R/P_2_, CAO1-F/R/P_5_, CAO1-F/R/P_6_, and CAO1-F/R/P_7_ was determined to be five copies of DNA. The sensitivity of three sets of CAO1-F/R/P_1_, CAO1-F/R/P_3_, and CAO1-F/R/P_4_ was determined to be ten copies of DNA. The sensitivity levels of all primer/probe sets in the range of 5–10 copies were similar to each other and also similar to that of the primer pair CAO1-F/R. It was concluded that the sensitivity of a PCR method was mostly determined by the primer pair and weakly affected by the probe.

### 3.4. Quantification of Blinded Samples by qPCR

Five *CAO1*-edited rice types (CAO1-2, CAO1-3, CAO1-4, CAO1-5, and CAO1-7) together with their primer/probe sets (CAO1-F/R/P_1_, CAO1-F/R/P_3_, CAO1-F/R/P_4_, CAO1-F/R/P_5_, and CAO1-F/R/P_7_) were used to evaluate the technical parameters of the editing-site-specific quantitative method. The genomic DNA from CAO1-2, CAO1-3, CAO1-4, CAO1-5, and CAO1-7 was serially diluted to the copy number concentrations covering four orders of magnitude, respectively (Supplementary Table S3). Using five diluted series of edited type DNA as templates, the standard curves of edited type DNA and the *PLD* gene were constructed by plotting the logarithm of the template copy number against the measured Ct values (Supplementary Figure S2). The technical parameters of correlation coefficient (R^2^), slope and amplification efficiency of standard curves are summarized in Supplementary Table S3. The R^2^ values of >0.99 revealed that there was a good linear relationship between Ct values and the logarithm for template copy number for both edited type DNA and the *PLD* gene. The slope values of the *PLD* standard curves were in the range of −3.546 to −3.332 and the slope values of edited type DNA standard curves ranged from −3.394 to −3.234. The amplification efficiencies of the *PLD* gene were estimated to be between 91.4% and 99.6%, and the amplification efficiencies of edited type DNA were estimated to be from 97.1% to 103.6% (Supplementary Table S3). The parameters of the standard curves of editing-site-specific PCR methods together with the *PLD* gene method were in the acceptable range, meeting the acceptance criteria for a qPCR method (European Commission, 2015).

Five sets of blinded samples were used to validate the accuracy of quantitative results by editing-site-specific qPCR. No positive signal for edited type DNA was observed for the five different editing-site-specific qPCR when detecting the lowest level sample of 0.1%, whereas the *PLD* gene qPCR produced normal amplification in these samples of 0.1%. According to the Ct values of blinded samples and their corresponding standard curves for edited type DNA as well as the *PLD* gene, the content of edited type DNA in blinded samples was calculated (Table 2). The relative standard deviation (RSD) and bias of measurement data were computed to evaluate the precision and trueness of quantitative results. According to the performance requirements for analytical methods of GMO testing, the precision in terms of RSD was defined to be ≤25%, and the trueness in terms of bias was defined to be within ±25% over the whole dynamic range [32]. For CAO1-2 specific qPCR, the experimental results of samples at 5% and 2% level displayed acceptable RSD and bias values, but the RSD value of the 1% sample was more than 25%. For CAO1-3, CAO1-4, and CAO1-5 specific qPCR, both RSD and bias values were within the acceptable range for the samples at 5% level; the RSD and/or bias values were beyond the acceptable range for the samples at the 2% and 1% levels. The RSD and bias values of all CAO1-7 samples exceeded the acceptable range by the CAO1-7 specific qPCR with an LNA-containing probe (Table 2). The 5% samples obtained relatively accurate quantitative results by qPCR, but with the decreasing amounts of edited type DNA content in their total DNA, the measured contents of the 2% and 1% samples were significantly lower than the expected values while the 0.1% samples failed to even generate amplification signals, and the variations in measurement results between replicates significantly increased.

The previous tests of sensitivity and dynamic range of editing-site-specific real-time PCR were performed using homozygous edited type DNA as templates; the detection sensitivity was determined to be less than 10 copies, and the lowest copy number within dynamic range was lower than 50 (Supplementary Table S3). Taking the sensitivity and dynamic range of editing-site-specific real-time PCR into account, the reason for the results was speculated to be that the competitive binding of the wild type DNA template to the universal primers influenced the amplification of edited type DNA in mixed samples, leading to underestimation or lack of amplification of edited type DNA in low-content samples. It was concluded that we had difficulty in obtaining accurate measurement results on the real-time PCR platform when the content of edited type DNA in the total DNA was less than 5%. The quantitative results demonstrated that the developed editing-site-specific qPCR was unfit to be applied for accurate quantification of the content of edited type DNA in mixed samples.

### 3.5. Quantification of Blinded Samples by ddPCR

The five sets of blinded samples were further quantified by ddPCR on the QX200 platform. Before carrying out quantification, the ddPCR assays were sufficiently optimized. The primer/probe concentrations were determined to be 400/200 nM and the annealing temperature was determined to be 55.5 °C, according to the fluorescent amplitude of positive droplets and the distinction between positive and negative droplets (Supplementary Figure S3). The same reaction condition was applied to the *PLD* gene since it was insensitive to variation of annealing temperature. In this study the total DNA was quantified by two methods of measuring the copy number of the *PLD* gene and measuring the copy number sum of edited type DNA and wild type DNA.

The edited type DNA and the *PLD* gene in blinded samples were simultaneously quantified by simplex ddPCR under the optimized conditions with homozygous edited DNA as the positive control, wild type DNA as the negative control, and ddH_2_O as the blank control. The amplification plots were shown in Supplementary Figure S4. The hot plots showed that the *PLD* gene amplified normally in all samples except the blank control. For editing-site-specific PCR assays, the negative control produced two droplet populations, one corresponding to droplets containing no template and the other corresponding to droplets containing wild type DNA. These two droplet populations were also observed in blinded samples that were made up of wild type DNA and edited type DNA. When analyzing the droplet readout results of blinded samples, the threshold must be automatically or manually set above the droplet populations of the negative control to eliminate the influence of unspecific amplification of wild type DNA. The distribution of positive droplets in blinded samples scattered in a wider range in comparison to the positive control. The presence of wild type DNA in positive droplets consumed the universal primers and affected the amplification of edited type DNA, leading to the lower signal amplitude of positive droplets. The dispersion of positive droplet clusters did not affect the distinction of positive droplets with the help of the negative control. The quantitative results of the blinded samples were calculated based on the measured copy number of edited type DNA and *PLD* gene (Table 3). The measured values of all blinded samples except the 0.1% CAO1-2 sample were very close to the expected values with acceptable RSD values from 0.42% to 12.3% and bias values from −16.66% to 13.86%; the RSD value of the quantitative results of 0.1% CAO1-2 sample was 55.6%, reflecting a larger variation between replicates. The quantitative results showed that the simplex ddPCR method can accurately quantify the content of genome-edited crops in samples at the level of 0.1% to 5%, meeting the performance requirements for GMO quantitative methods.

The copy numbers of edited type DNA and wild type DNA for each sample were simultaneously quantified in the same single reaction tube by duplex ddPCR containing a universal primer pair, the edited type probe labeled with FAM, and the wild type probe labeled with HEX. The one-dimensional (1-D) and two-dimensional (2-D) hot plots are shown in Supplementary Figures S5 and S6. The 1-D hot plots showed that the primer/probe set of CAO1-5 amplified two negative signal clusters from wild type DNA, and the primer/probe set of wild type rice generated two negative signal clusters from the edited type DNA of CAO1-2, CAO1-3, CAO1-5, and CAO1-7 (Supplementary Figure S5). Compared to the hot plots of simplex ddPCR (Supplementary Figure S4), four edited type primer/probe sets only generated one negative signal cluster from the wild type DNA. The reason for this was speculated to be that the difference in signal intensity between droplets containing wild type DNA and containing no DNA was so small that two clusters overlapped to form a single cluster. This explanation is also suitable to the amplification of the wild type primer/probe set with edited type DNA as a template. For the duplex ddPCR, the edited type primer/probe sets used wild type DNA as the negative control and corresponding edited type DNA as the positive control; by contrast, the wild type primer/probe set used edited type DNA as the negative control and wild type DNA as the positive control. The threshold of blinded samples should be automatically or manually set above the signal clusters of the negative control to eliminate the influence of unspecific amplification. The total DNA amount was calculated to be the sum of edited type DNA and wild type DNA. The quantitative results of blinded samples are shown in Table 3. The measured ratio values of all blinded samples were very close to the expected values with acceptable RSD values from 0.31% to 9.18 and bias values from −9.69% to 6.43%. The quantitative results showed that the unspecific amplification of the wild type primer/probe set did not affect the quantification of wild type DNA sequence by setting edited type DNA as the negative control. The quantitative method by duplex ddPCR was demonstrated to also be able to accurately quantify the genome-edited ingredient content at the level of 0.1% to 5%, meeting the performance requirements for GMO quantitative methods.

Two calculation methods for genome-edited ingredient content were evaluated by simplex ddPCR and duplex ddPCR, respectively. Compared to the data by simplex ddPCR, the measurement data from duplex ddPCR fluctuated in a narrower range with smaller RSD values and was much closer to the expected values with a narrower bias interval (Table 3). The duplex ddPCR displayed higher precision and trueness than simplex ddPCR. A t-test was performed to analyze the difference in the two sets of measurement data given by two different calculation methods for the same blinded sample. The result was that no significant difference was observed between the two calculation methods with *p* values of more than 0.5. Both calculation methods obtained accurate and comparable quantitative results for all blinded samples, which demonstrated that the total DNA amount can be expressed as the copy number of a taxon-specific single-copy reference gene and also as the sum of edited type DNA and wild type DNA copy numbers. The content of genome-edited rice can be accurately quantified by using the developed editing-site-specific PCR method on the ddPCR platform, and it can be calculated by the two ways: CE (%) = copy number of edited type DNA/copy number of *PLD* gene*100, or CE (%) = copy number of edited type DNA/(copy number of edited type DNA + copy number of wild type DNA) × 100.

## 4. Discussion

An editing-site-specific PCR method was proposed and validated for detection of genome-edited plants with *CAO1*-edited rice as materials. In the present case, the mutated bases were incorporated into a probe sequence to design a probe specific to the genome-edited region. Incorporation of mutated bases into primer sequences would be an alternative strategy in designing an editing-site-specific method. In order to ensure the specificity of the PCR method, the mutated bases must be placed at the 3′ end of the primer [11]. Therefore, this strategy strictly fixes the position in nucleotide sequences where the primer should be located. Fixing the position of primers in DNA sequences is likely to result in the failure to design a suitable primer pair with acceptable sensitivity. This strategy of incorporating mutated bases into probe sequences relies on the specific recognition of the probe to the edited sequence to identify the specific genome-edited plant. To verify the feasibility and applicability of this strategy, a total of eight TaqMan probes were designed to correspond to one wild type rice and seven edited type rice. The presence of a single-nucleotide mutation in the probe was demonstrated to not be enough to recognize its corresponding target sequence. For instance, the primer/probe set of CAO1-F/R/P_7_ of CAO1-7 produced unspecific amplification from wild type DNA (Figure 2h). The use of LNAs in probe design efficiently increased the specificity of the primer/probe system and achieved successful identification of genome-edited rice harboring a single-nucleotide insertion. The seven primer/probe sets designed for the editing-site-specific PCR method exhibited high detection specificity and sensitivity. Based on the identification result, the genome-edited ingredient can be traceable to a specific genome-edited product and its developer.

The use of a primer pair universal to edited-type DNA and wild-type DNA resulted in the competitive binding of wild-type DNA to the universal primer during the reaction, thus leading to underestimation of the genome-edited ingredient content in mixed samples on the real-time PCR platform. The ddPCR platform partitions the reaction mixture into thousands of water-in-oil microdroplets, each droplet containing little to no wild type DNA [33,34]. Resultantly, the competitive consumption of universal primers by wild type DNA was greatly reduced, and accurate quantitative results of blinded samples were obtained on the ddPCR platform. Digital PCR has been the mainstream technology for absolute quantification of nucleic acid without dependence on reference materials and standard curves, it has been widely used for reference material characterization, GMO quantification, and gene-editing frequency evaluation [30,31,35,36,37]. Digital PCR technology will definitely be used for quantification of genome-edited plants.

The genome-edited ingredient content is calculated as the ratio of the edited type DNA copy number to the total DNA copy number by ddPCR; correct measurement of the total DNA copy number is crucial for accurately quantifying the genome-edited ingredient content. This research proposed an alternative method for measuring the total DNA amount that can be expressed as the copy number sum of wild type DNA and edited type DNA. When adopting this strategy, the reference gene is not necessary for characterizing the total DNA amount. This newly presented strategy is suitable for the quantitative detection of single-copy genes in diploid crops such as rice, corn, and soybean. Just like when using a taxon-specific reference gene to measure the total DNA copy number, both the copy number of the target gene in a haploid genome and the genome ploidy of a test crop must be considered [38]). Whether a taxon-specific reference gene or an edited target gene is used to characterize the total DNA amount of polyploid crops, such as rapeseed and wheat, the copy number of total DNA must be determined according to the gene copy number in haploid and genome ploidy.

The seven *CAO1*-edited rice plants used in this research were proved to be homozygotes by Sanger sequencing. During optimization of the ddPCR conditions, we found that the copy number ratio of the edited type DNA and the *PLD* gene was approximate 0.5 for the two *CAO1*-edited plants of CAO1-1 and CAO1-6 (Supplementary Table S4). It was speculated that a deletion of a large fragment homologous to the edited region may have happened to these two genome-edited plants, a situation identical to what has also been found in previous research [39,40]. Since the genotype of these two *CAO1*-edited plants was uncertain, we gave up using these two plants for subsequent validation of quantitative methods. The data of Sanger sequencing is not enough to determine the genotype of genome-edited plants and so the genotype of genome-edited plants is best further confirmed based on the dPCR results or the progeny segregation after self-fertilization.

This study overcame the drawback of the previously developed methods, and established an editing-site-specific PCR method for identification and quantification of genome-edited plants with good applicability and practicability. The application of this method is compatible with the existing equipment, reagents, basic operation, and molecular biology expertise found in GMO testing laboratories. The performance of this method is identical to standard qPCR methodology with sufficient specificity, sensitivity, and dynamic range. The validation results of blinded samples demonstrated that this approach is able to quantify the genome-edited ingredients on the ddPCR platform with satisfactory precision and trueness, meeting the GMO regulatory requirements and performance requirements for GMO detection methods. The developed editing-site-specific PCR method presents a promising detection and quantification technique for analysis-based regulation of genome-edited plants that is fully compliant with current GMO regulations. However, the method only can be applied for detection of genome-edited products with known information since it was developed based on the known edited sequence. The modification introduced by gene editing technology might be indistinguishable from random mutations of naturally occurring or induced by chemicals and irradiation, a database disclosing the information on genome-edited plants should be set up to provide data support for developing analytical methods and monitoring genome-edited plants [27]. It is difficult to trace and identify genome-edited food and feed products without relevant information, and to screen genome-edited products with unknown information would be a huge challenge faced by the surveillance of genome-edited plants.

## Figures and Tables

**Table 1:** Information of used primers and probes.

Target	Name	Sequence (5→3′)	Amplicon Size (bp)
*PLD* gene	KVM159	TGGTGAGCGTTTTGCAGTCT	68
	KVM160	CTGATCCACTAGCAGGAGGTCC	
	TM013	TGTTGTGCTGCCAATGTGGCCTG ^a^	
*CAO1* gene	CAO1-F	CGGTGATGATGGAGCTGACT	123–144
	CAO1-R	GATATCGAACCGGACCACCT	
CAO1-w	CAO1-P_W_	TGCAATTGGAACCCTTGGCCC ^b^	142
CAO1-1	CAO1-P_1_	AAGAACTTGCCTTTTCCCCGG ^a^	125
CAO1-2	CAO1-P_2_	TTGGAACCCTTGGAACCCCG ^a^	144
CAO1-3	CAO1-P_3_	CCCTTGCCCGGGTCATC ^a^	140
CAO1-4	CAO1-P_4_	CAATTGGAACCCTTCCCCGG ^a^	140
CAO1-5	CAO1-P_5_	AGGCCTTGTTCCCATTGCAAA ^a^	123
CAO1-6	CAO1-P_6_	CCATGTCGATGACGTTCCAATTGC ^a^	130
CAO1-7	CAO1-P_7_	TTGGAACCCTTGGT * CCCCG ^a^	143

Superscript * representing the locked nucleic acid (LNA) of T, ^a^ indicating the probe labeled with FAM, ^b^ indicating the probe labeled with FAM and HEX.

**Table 2:** Statistical analysis of quantitative results of blinded samples by editing-site-specific qPCR.

Sample	Theoretical (%)	Experimental (%)			Mean Experiment (%)	SD	RSD (%)	Bias (%)
		1	2	3				
CAO1-2	5	5.74	4.99	5.23	5.32	0.39	7.25	6.42
	2	2.29	2.00	1.73	2.01	0.28	13.78	0.37
	1	1.37	0.95	0.76	1.03	0.32	30.83	2.58
CAO1-3	5	6.42	5.89	4.11	5.47	1.21	22.15	9.47
	2	2.15	1.71	1.11	1.66	0.52	31.56	−17.07
	1	0.93	0.82	0.14	0.63	0.43	68.25	−37.20
CAO1-4	5	5.31	5.13	4.07	4.84	0.67	13.84	−3.22
	2	2.02	1.69	0.96	1.56	0.54	34.88	−22.08
	1	0.49	0.45	0.05	0.33	0.24	73.31	−66.81
CAO1-5	5	4.62	4.51	4.60	4.58	0.06	1.24	−8.45
	2	1.12	0.95	0.94	1.00	0.10	10.23	−49.85
	1	0.22	0.21	0.22	0.22	0.005	2.11	−78.23
	5	2.47	2.24	2.30	2.34	0.12	5.16	−53.27
CAO1-7	2	0.21	0.10	0.06	0.12	0.08	60.24	−93.77
	1	0.06	0.02	0.02	0.03	0.02	61.03	−96.72

**Table 3:** Statistical analysis of quantitative results of blinded samples by ddPCR.

Sample	Expected Value (%)	Experimental Data (%) by Simplex ddPCR (Edited Type DNA Copy Number/PLD Gene Copy Number)							Experimental Data (%) by Duplex ddPCR (Edited Type DNA Copy Number/Sum of Edited Type and Wild Type DNA Copy Number)						
		1	2	3	Mean	SD	RSD (%)	Bias (%)	1	2	3	Mean	SD	RSD (%)	Bias (%)
CAO1-2	5	4.91	4.66	4.67	4.75	0.14	2.99	−5.06	4.78	4.75	4.90	4.81	0.08	1.63	−3.79
	2	1.93	1.78	1.64	1.78	0.15	8.25	−10.95	2.04	1.98	1.94	1.99	0.05	2.37	−0.63
	1	0.99	0.95	0.95	0.96	0.03	2.63	−3.71	0.93	0.92	0.99	0.95	0.04	4.08	−5.42
	0.1	0.15	0.1	0.04	0.1	0.06	55.66	−0.6	0.09	0.09	0.10	0.09	0.01	9.18	−6.37
CAO1-3	5	5.1	4.87	4.88	4.95	0.13	2.55	−1.01	5.22	5.12	4.84	5.06	0.20	3.91	1.15
	2	2.12	2.03	1.85	2.00	0.13	6.65	−0.04	2.15	2.10	2.13	2.13	0.02	1.08	6.43
	1	1.05	1.04	0.99	1.03	0.03	2.82	2.81	1.00	1.00	1.00	1.00	0.00	0.31	−0.09
	0.1	0.1	0.09	0.09	0.09	0.01	6.43	−10.06	0.10	0.10	0.11	0.10	0.00	4.64	0.21
CAO1-4	5	4.51	4.43	4.31	4.41	0.1	2.29	−11.71	5.04	5.16	5.19	5.13	0.08	1.57	2.54
	2	1.99	1.9	1.72	1.87	0.14	7.4	−6.6	2.17	1.87	2.02	2.02	0.15	7.48	1.00
	1	1.03	0.98	0.89	0.97	0.07	7.24	−2.97	1.01	0.90	1.01	0.97	0.07	6.75	−2.93
	0.1	0.1	0.08	0.08	0.08	0.01	12.3	−16.66	0.10	0.09	0.09	0.09	0.01	6.03	−9.69
CAO1-5	5	4.74	4.86	4.84	4.81	0.06	1.28	−3.72	5.02	4.77	5.03	4.94	0.14	2.93	−1.17
	2	1.92	1.92	1.81	1.88	0.07	3.56	−5.84	2.01	1.95	1.99	1.98	0.03	1.66	−0.85
	1	0.97	0.96	0.96	0.96	0.01	0.65	−3.77	1.10	0.98	0.95	1.01	0.08	7.87	1.25
	0.1	0.12	0.1	0.12	0.11	0.01	10.32	13.86	0.09	0.11	0.10	0.10	0.01	10.10	−0.21
	5	4.70	4.79	4.73	4.74	0.05	1.04	−5.19	4.96	5.02	4.95	4.98	0.04	0.76	−0.48
CAO1-7	2	2.06	2.05	2.06	2.05	0.01	0.42	2.74	2.16	1.92	2.05	2.04	0.12	5.67	2.13
	1	0.90	0.95	0.90	0.92	0.03	2.76	−8.41	0.99	1.09	1.08	1.05	0.05	5.05	5.10
	0.1	0.10	0.09	0.09	0.10	0.01	5.46	−4.19	0.11	0.10	0.10	0.11	0.01	7.45	5.19

## Supplementary Materials

The following are available online at https://www.mdpi.com/article/10.3390/foods10061209/s1, Table S1. Three primer pairs designed for screening optimal primer pair, Table S2. Sensitivity test of editing-site-specific PCR method, Table S3. Parameters of standard curves of editing-site-specific and PLD gene PCR methods with diluted edited type DNA as templates, Table S4. Measured copy number ratio of edited type DNA and PLD gene for CAO1-edited rice CAO1-1 and CAO1-6 by ddPCR, Figure S1. Screening of the universal primer pair with wild type rice DNA as template, Figure S2. Amplification plots together with standard curves of PLD gene and edited type DNA using serially diluted edited type DNA as templates, Figure S3. Optimization of annealing temperature and primer/probe concentration of ddPCR, Figure S4. Amplification plots of edited type DNA and PLD gene by simplex ddPCR with blinded samples and controls as templates, Figure S5. One-dimensional amplification plots of duplex ddPCR of editing/wild type DNA, Figure S6. Two-dimensional hot plots of duplex ddPCR of edited/wild type DNA.
