# Supplementary material for: An Editing-Site-Specific PCR Method for Detection and Quantification of CAO1-Edited Rice
Source: Foods. 2021 May 27;10(6):1209. doi: 10.3390/foods10061209 (PMC8226746; doi:10.3390/foods10061209)
Supplement: Supplementary file 1 [file foods-10-01209-s001.zip › foods-1221815-supplementary.pdf]

# Supplementary Material

## An editing-site-specific PCR method for detection and quantification of *CAO1*-edited rice

Hongwen Zhang, Jun Li, Shenbo Zhao, Xiaohong Yan, Nengwu Si, Hongfei Gao, Yunjing Li, Shanshan Zhai, Fang Xiao, Gang Wu *, Yuhua Wu *

Key Laboratory of Biology and genetic improvement of oil crops of the Ministry of Agriculture and Rural Affairs, Oil Crops Research Institute, Chinese Academy of Agricultural Sciences, Wuhan 430062, China

* Correspondence: Wu G., wugang@caas.cn; Tel.: +86-27-86711501. Wu Y. H., wuyuhua@oilcrops.cn; Tel.: +86-27-86711573

Table S1 Three primer pairs designed for screening optimal primer pair

| Primer | Sequence (5’-3’) | Amplicon size (bp) |
|---|---|---|
| CAO1-$F_1$ | CGGTGATGATGGAGCTGACT | 142 |
| CAO1-$R_1$ | GATATCGAACCGGACCACCT | |
| CAO1-$F_2$ | GGTGTTGGTAGATATGGAGGAATCA | 204 |
| CAO1-$R_2$ | CGCCCTCCAATCGCAATA | |
| CAO1-$F_3$ | TGATGGAGCTGACTATGCAAAG | 139 |
| CAO1-$R_3$ | CTGGATATCGAACCGGACC | |

Table S2 Sensitivity test of editing-site-specific PCR method

| Primer/probe set | Template | Copy number | signal ration | Mean of Ct values | SD | RSD |
|---|---|---|---|---|---|---|
| CAO1-F/R/$P_w$ | CAO1-W | 80 | 4/4 | 34.00 | 0.0 | 0.23 |
| | | 40 | 4/4 | 34.78 | 0.2 | 0.82 |
| | | 20 | 4/4 | 36.36 | 0.6 | 1.88 |
| | | 10 | 4/4 | 37.45 | 0.5 | 1.56 |
| | | 5 | 10/10 | 38.96 | 0.6 | 1.66 |
| | | 1 | 1/10 | / | | |
| CAO1-F/R/$P_1$ | CAO1-1 | 80 | 4/4 | 34.63 | 0.0 | 0.11 |
| | | 40 | 4/4 | 35.75 | 0.1 | 0.33 |
| | | 20 | 4/4 | 36.26 | 0.2 | 0.66 |
| | | 10 | 4/4 | 37.46 | 0.2 | 0.75 |
| | | 5 | 9/10 | / | 0.9 | 2.47 |
| | | 1 | 1/10 | / | | |
| CAO1-F/R/$P_2$ | CAO1-2 | 80 | 4/4 | 33.06 | 0.1 | 0.45 |
| | | 40 | 4/4 | 34.11 | 0.1 | 0.56 |
| | | 20 | 4/4 | 35.18 | 0.2 | 0.84 |
| | | 10 | 4/4 | 35.63 | 0.1 | 0.38 |
| | | 5 | 10/10 | / | 0.7 | 2.12 |
| | | 1 | 1/10 | / | | |
| CAO1-F/R/$P_3$ | CAO1-3 | 80 | 4/4 | 34.16 | 0.0 | 0.19 |
| | | 40 | 4/4 | 35.06 | 0.1 | 0.31 |
| | | 20 | 4/4 | 36.51 | 0.2 | 0.69 |
| | | 10 | 4/4 | 38.06 | 0.0 | 0.23 |
| | | 5 | 7/10 | / | 0.7 | 1.95 |
| | | 1 | 2/10 | / | 0.3 | 0.91 |
| CAO1-F/R/$P_4$ | CAO1-4 | 80 | 4/4 | 34.25 | 0.0 | 0.20 |
| | | 40 | 4/4 | 35.35 | 0.1 | 0.33 |
| | | 20 | 4/4 | 36.61 | 0.2 | 0.76 |
| | | 10 | 4/4 | 37.39 | 0.2 | 0.77 |
| | | 5 | 9/10 | / | 1.1 | 2.78 |
| | | 1 | 1/10 | / | | |
| CAO1-F/R/$P_5$ | CAO1-5 | 80 | 4/4 | 34.03 | 0.0 | 0.28 |
| | | 40 | 4/4 | 35.02 | 0.1 | 0.54 |
| | | 20 | 4/4 | 36.16 | 0.2 | 0.61 |
| | | 10 | 4/4 | 37.25 | 0.4 | 1.24 |
| | | 5 | 10/10 | / | 0.8 | 2.12 |
| | | 1 | 1/10 | / | | |
| CAO1-F/R/$P_6$ | CAO1-6 | 80 | 4/4 | 33.13 | 0.0 | 0.14 |
| | | 40 | 4/4 | 34.27 | 0.1 | 0.55 |
| | | 20 | 4/4 | 35.57 | 0.0 | 0.25 |

| | | | | | | |
|---|---|---|---|---|---|---|
| | | 10 | 4/4 | 37.22 | 0.1 | 0.42 |
| | | 5 | 10/10 | 37.92 | 0.8 | 2.31 |
| | | 1 | 2/10 | / | 0.0 | 0.19 |
| CAO1-F/R/$P_7$ | CAO1-7 | 80 | 4/4 | 34.28 | 0.1 | 0.37 |
| | | 40 | 4/4 | 35.50 | 0.1 | 0.36 |
| | | 20 | 4/4 | 36.42 | 0.0 | 0.19 |
| | | 10 | 4/4 | 37.70 | 0.0 | 0.25 |
| | | 5 | 10/10 | 38.64 | 0.5 | 1.31 |
| | | 1 | 1/10 | / | | |

Table S3 Parameters of standard curves of editing-site-specific and *PLD* gene PCR methods with diluted edited type DNA as templates

| Genome -edited rice | Calibrant concentration (copies/μL) | Primer/probe set | Parameters of standard curves | | |
|---|---|---|---|---|---|
| | | | $R^2$ | Slope | Amplification efficiency (%) |
| CAO1-2 | 258600, 25860, | CAO1-F/R/$P_2$ | 0.998 | -3.390 | 97.3 |
| | 2586, 259, 26 | KVM159/KVM160/TM013 | 0.998 | -3.346 | 99.0 |
| CAO1-3 | 237500, 23750, | CAO1-F/R/$P_3$ | 0.998 | -3.293 | 101.2 |
| | 2375, 238, 24 | KVM159/KVM160/TM013 | 0.999 | -3.466 | 94.3 |
| CAO1-4 | 231100, 23110, | CAO1-F/R/$P_4$ | 0.998 | -3.366 | 98.1 |
| | 2311, 231, 23 | KVM159/KVM160/TM013 | 0.999 | -3.343 | 99.1 |
| CAO1-5 | 390300, 39030, | CAO1-F/R/$P_5$ | 0.999 | -3.239 | 103.6 |
| | 3903, 390, 39 | KVM159/KVM160/TM013 | 0.999 | -3.332 | 99.6 |
| CAO1-7 | 362600, 36260, | CAO1-F/R/$P_7$ | 0.998 | -3.394 | 97.1 |
| | 3626, 363, 36 | KVM159/KVM160/TM013 | 0.998 | -3.546 | 91.4 |

Table S4 Measured copy number ratio of edited type DNA and *PLD* gene for *CAO1*-edited rice CAO1-1 and CAO1-6 by ddPCR

| Name | Gene | Measured copy number | | | Ratio (%) |
|---|---|---|---|---|---|
| | | 1 | 2 | Mean | |
| CAO1-1 | Editing DNA | 415 | 405 | 410 | 50.96 |
| | *PLD* | 808 | 801 | 804.5 | |
| CAO1-6 | Editing DNA | 361 | 355 | 358 | 50.82 |
| | *PLD* | 667 | 742 | 704.5 | |

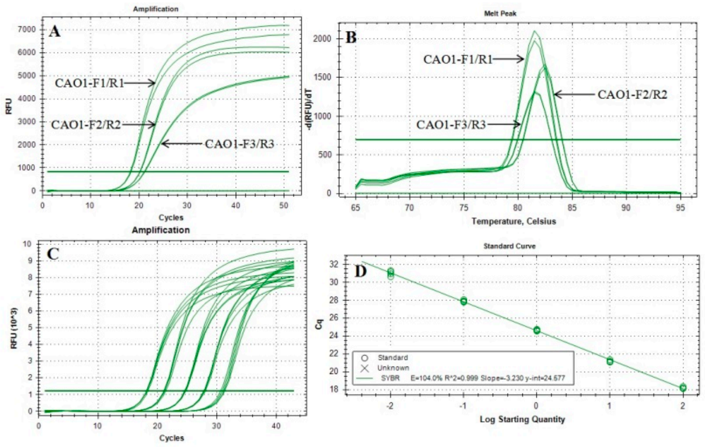


Fig. S1 Screening of the universal primer pair with wild type rice DNA as template. A, the amplification plots of the three primer pairs of CAO1-F1/R1, CAO1-F2/R2 and CAO1-F3/R3; B, the melting curves of the three primer pairs of CAO1-F1/R1, CAO1-F2/R2 and CAO1-F3/R3; C, the amplification plots of primer pair CAO1-F1/R1 with serial dilutions of wild type DNA as templates. D, The standard curve constructed by plotting measured Ct values against the logarithm of template copy number.

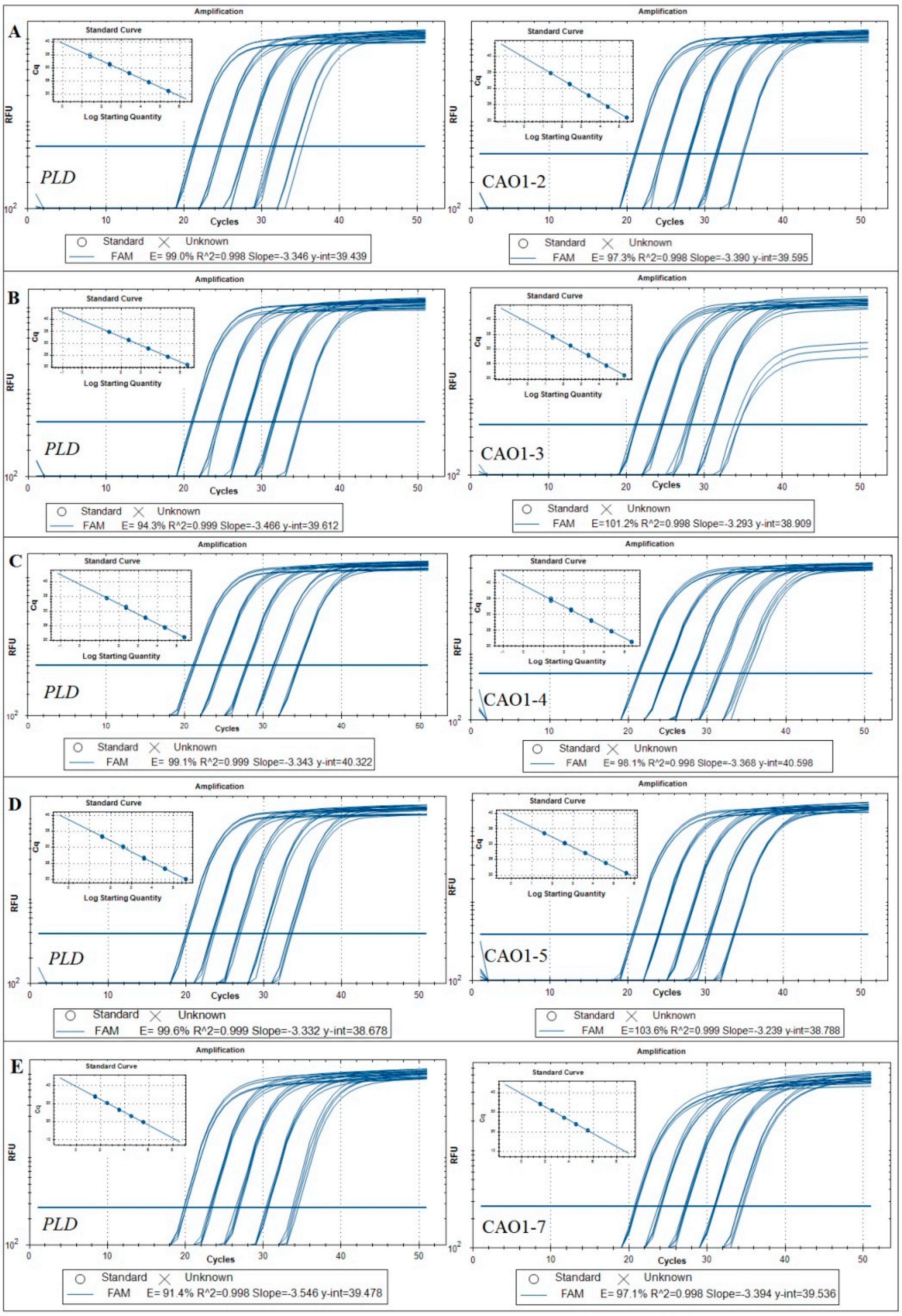


Fig. S2 Amplification plots together with standard curves of *PLD* gene and edited type DNA using serially diluted edited type DNA as templates. A, the amplification plots and standard curves of *PLD* gene and edited sequence of CAO1-2 using serial dilutions of CAO1-2 DNA as templates; B, the amplification plots and standard curves of *PLD* gene and edited sequence of CAO1-3 using serial dilutions of CAO1-3 DNA as templates; C, the amplification plots and standard curves of *PLD* gene and edited sequence of CAO1-4 using serial dilutions of CAO1-4 DNA as templates; D, the amplification plots and standard curves of *PLD* gene and edited sequence of CAO1-5 using serial dilutions of CAO1-5 DNA as templates; E, the amplification plots and standard curves of *PLD* gene and edited sequence of CAO1-7 using serial dilutions of CAO1-7 DNA as templates.

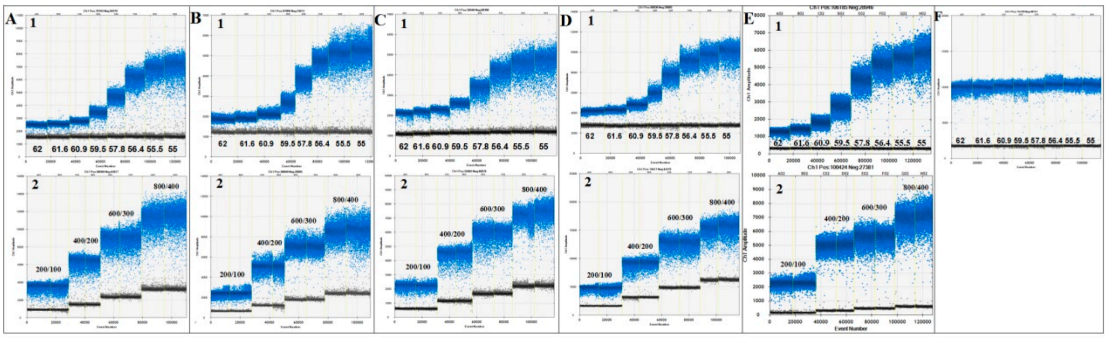

Fig. S3 Optimization of annealing temperature and primer/probe concentration of ddPCR. A-Z showed the optimization of ddPCR of CAO1-2, CAO1-3, CAO1-4, CAO1-5 and CAO1-7, respectively; F showed the optimization of annealing temperature of *PLD* ddPCR. The five primer/probe sets exhibited similar amplification pattern under different reaction conditions. Number "1" showed the optimization of annealing temperature, the temperature gradient was set from 55°C to 62°C, the signal amplitude of positive droplets for the five editing-site-specific ddPCR raised with the decrease of temperature, and 55.5℃ was determined to be the optimal annealing temperature of ddPCR. Number "2" showed the optimization of primer/probe concentration, four concentration gradients of 200/100 nM, 400/200 nM, 600/300 nM and 800/400 nM were set for the five editing-site-specific ddPCR, the signal amplitude of positive droplets for the five ddPCR raised with the increase of primer/probe concentration, and the concentration of 400/200 nM identical to the primer/probe concentration for real-time PCR, was selected for ddPCR.

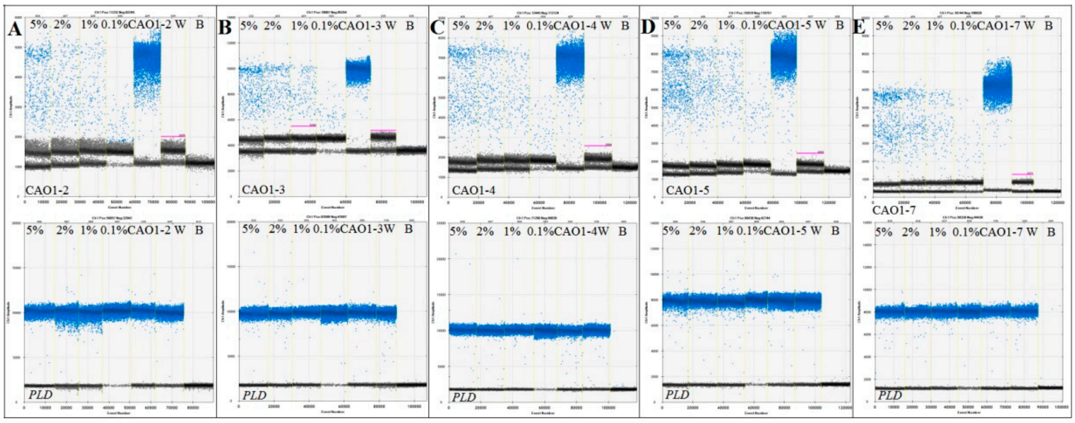

Fig. S4 Amplification plots of edited type DNA and *PLD* gene by simplex ddPCR with blinded samples and controls as templates. A-E showed the amplification plots of blinded samples of CAO1-2, CAO1-3, CAO1-4, CAO1-5 and Cao1-7, as well as positive control, negative control and blank control. The blinded samples included four levels of 5%, 2%, 1% and 0.1%; the pure DNA of CAO1-2, CAO1-3, CAO1-4, CAO1-5 and Cao1-7 was used as positive control, respectively; W represented wild type DNA used as negative control; B represented blank control.

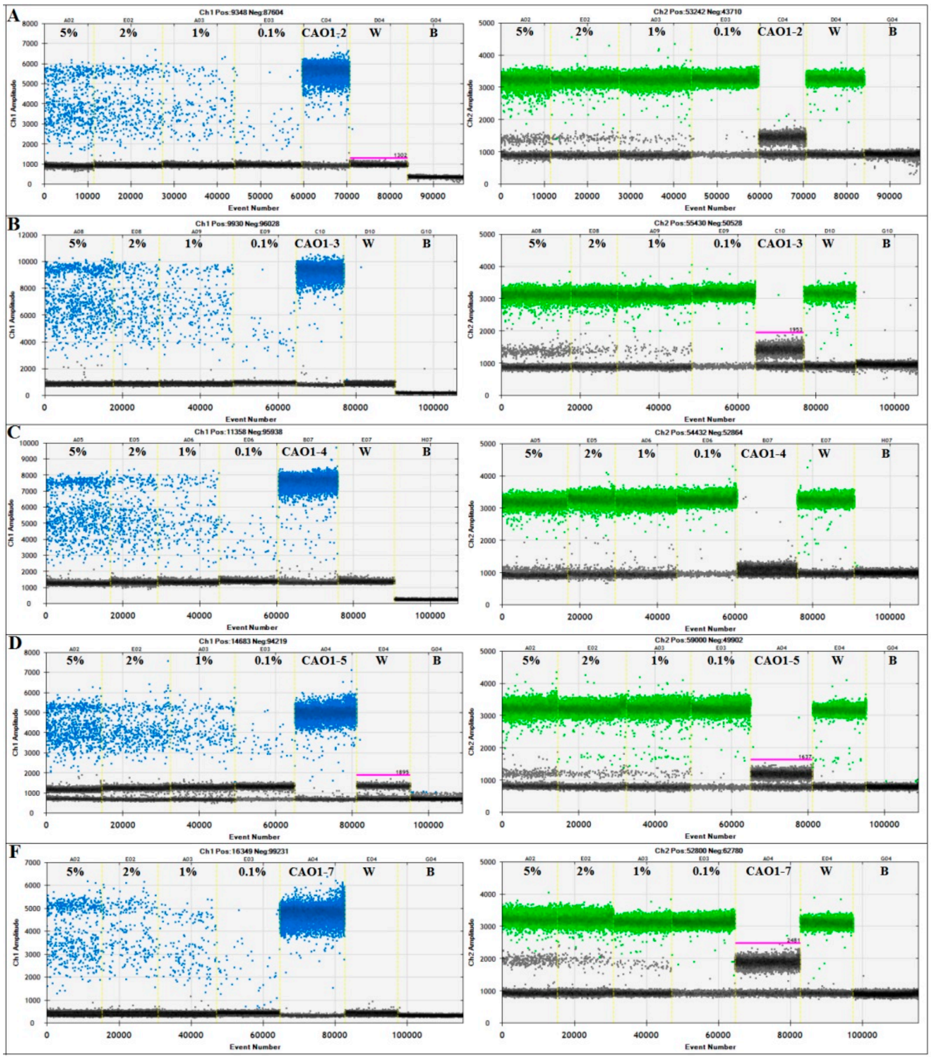


Fig. S5 One-dimensional amplification plots of duplex ddPCR of editing/wild type DNA. The edited type probe labeled with FAM produced blue positive droplets; the wild type probe labeled with HEX produced green positive droplets. A-E showed the one-dimension hot plots of blinded samples of CAO1-2, CAO1-3, CAO1-4, CAO1-5 and CAO1-7, as well as positive control, negative control and blank control. The blinded samples of each CAO1-edited rice plant included four levels of 5%, 2%, 1%, and 0.1%. For the amplification of edited type DNA in blinded samples, the pure DNA of CAO1-2, CAO1-3, CAO1-4, CAO1-5 and CAO1-7 was used as positive control, respectively; the wild type DNA expressed as W was used as negative control; the water expressed as B was used as blank control; by contrast, for the amplification of wild type DNA in blinded samples, the pure DNA of CAO1-2, CAO1-3, CAO1-4, CAO1-5 and CAO1-7 was used as negative control, respectively; the wild type DNA expressed as W was used as positive control.

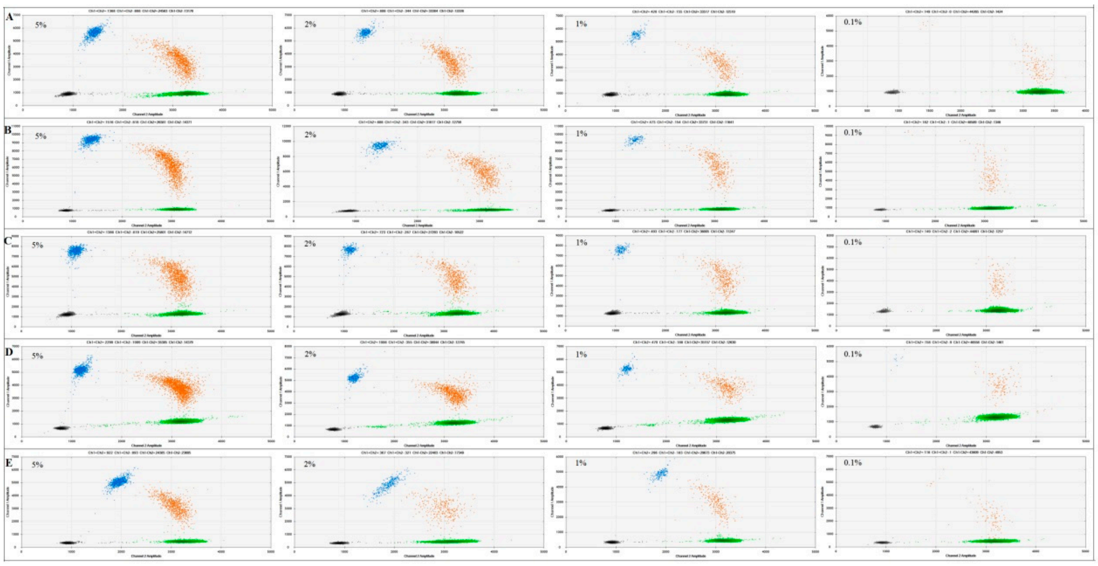

Fig. S6 Two-dimensional hot plots of duplex ddPCR of edited/wild type DNA. A-E showed the two-dimensional hot plots of blinded samples of CAO1-2, CAO1-3, CAO1-4, CAO1-5 and CAO1-7, respectively. Each set of blinded samples included four levels of 5%, 2%, 1% and 0.1%. For each blinded sample four droplet clusters were observed, corresponding to negative droplets (grey), FAM-positive droplets (blue), HEX-positive droplets (green), dual fluorophore (FAM and HEX)-positive droplets (orange).
